# Impact of Anterior Mitral Leaflet Length on the Efficacy of Intracardiac Echocardiography-Guided Endocardial Septal Ablation for HOCM

**DOI:** 10.3390/jcdd13060274

**Published:** 2026-06-16

**Authors:** Yunlong Ling, Tao Yu, Ye Xu, Quan Wan, Yang Pang, Guijian Liu, Chaofeng Chen, Kuan Cheng, Wenqing Zhu, Qingxing Chen, Junbo Ge

**Affiliations:** 1 Department of Cardiology, Shanghai Institute of Cardiovascular Diseases, Zhongshan Hospital, Fudan University, Shanghai 200032, China; ling.yunlong@zs-hospital.sh.cn (Y.L.); 23111210146@m.fudan.edu.cn (T.Y.); xu.ye@zs-hospital.sh.cn (Y.X.); 851017py@163.com (Y.P.); liu.guijian@zs-hospital.sh.cn (G.L.); ccffdzsyy3@163.com (C.C.); cheng.kuan@zs-hospital.sh.cn (K.C.); ge.junbo2@zs-hospital.sh.cn (J.G.); 2 Department of Echocardiography, Zhongshan Hospital, Fudan University, Shanghai 200032, China; wan.quan@zs-hospital.sh.cn

**Keywords:** hypertrophic obstructive cardiomyopathy, percutaneous endocardial septal ablation, intracardiac echocardiography, anterior mitral leaflet length, left ventricular outflow tract obstruction

## Abstract

**Background:** Intracardiac echocardiography (ICE)-guided percutaneous endocardial septal ablation (PESA) is a promising alternative strategy for symptomatic hypertrophic obstructive cardiomyopathy (HOCM). However, the extent of left ventricular outflow tract gradient (LVOTG) reduction after PESA varies considerably among patients, and reliable echocardiographic predictors of procedural efficacy remain unclear. We aimed to identify echocardiographic predictors of procedural efficacy and to describe our ablation strategy. **Methods:** We retrospectively analyzed 30 consecutive symptomatic HOCM patients who underwent ICE-guided PESA. The primary endpoint was the absolute change in resting LVOTG from baseline to the 1-year follow-up; a key secondary endpoint was significant gradient reduction, defined as a ≥50% decrease in resting LVOTG. Associations between LVOTG reduction and baseline echocardiographic parameters, particularly anterior mitral leaflet length (AMLL) and interventricular septal thickness (IVST), were evaluated. **Results:** At 1 year, the mean resting LVOTG decreased from 86.03 ± 24.30 mmHg to 41.43 ± 18.49 mmHg (*p* < 0.001). For the key secondary endpoint (≥50% resting LVOTG reduction), AMLL was the only variable associated with the outcome in the prespecified multivariable logistic model (OR per 1 mm increase: 0.429; *p* = 0.006), with consistent findings in a Firth penalized logistic regression sensitivity analysis. ROC analysis identified an exploratory AMLL cutoff of approximately 29 mm (AUC 0.920; sensitivity 90%; specificity 80%). In the continuous-outcome analysis, each 1 mm increase in AMLL corresponded to approximately 4.3 mmHg less LVOTG reduction (β = −4.32; *p* < 0.001). Thinner IVST was not significantly associated with greater procedural efficacy (*p* = 0.052), suggesting a potential thickness-dependent limit that warrants further investigation. **Conclusions:** ICE-guided PESA effectively reduces LVOT obstruction in HOCM. Shorter AMLL predicted greater LVOT gradient reduction in this exploratory cohort, and a Youden-derived AMLL cutoff of approximately 29 mm may help identify patients who are more likely to achieve substantial gradient reduction.

## 1. Introduction

Hypertrophic cardiomyopathy (HCM) is a genetic disorder characterized by heterogeneous clinical presentations, primarily driven by mutations in sarcomere proteins. Left ventricular outflow tract obstruction (LVOTO) is a hallmark of the disease, present in the majority of patients at rest or during provocation. For patients with a resting or provoked gradient ≥ 50 mmHg who remain symptomatic despite optimal medical therapy, therapies aimed at reducing LVOTO and the gradient are indicated [1].

While surgical myectomy remains the gold standard, percutaneous options are essential for patients at high surgical risk or with variable anatomy. Since Cooper et al. [2] first reported ICE-guided endocardial septal radiofrequency ablation in 2016, the technique has been increasingly adopted due to its precision and independence from coronary anatomy.

Although the short-term and mid-term efficacy of PESA has been demonstrated, inter-patient variability in gradient reduction exists [3,4,5]. The mechanism of LVOTO involves both septal hypertrophy and systolic anterior motion (SAM) of the mitral valve [6]. The severity of obstruction is governed by a complex interplay between septal hypertrophy, mitral valve apparatus anatomy (e.g., leaflet length), and flow dynamics (Venturi vs. drag forces). Specifically, an elongated anterior mitral leaflet (AMLL) is a known phenotypic trait in HOCM that exacerbates obstruction [7].

Historically, the mechanism of systolic anterior motion (SAM) was primarily attributed to the Venturi effect—a suction force generated by high-velocity blood flow in the narrowed outflow tract [8]. However, contemporary hemodynamic studies suggest that hydrodynamic drag forces, rather than the Venturi effect alone, play a dominant role. The ejection flow impinges on the anterior mitral leaflet, driving it toward the septum and thereby sustaining SAM. In this model, the mitral leaflet is not merely sucked in but is actively pushed into the septum by the flow stream, engaging its protruding surface. This paradigm shift underscores the critical role of anterior mitral leaflet length (AMLL). An elongated leaflet protrudes further into the path of ejection, creating a larger surface area for drag forces to act upon [9,10]. Current HCM guidelines recognize a markedly elongated anterior mitral leaflet as a condition that may warrant concomitant surgical mitral valve intervention during septal myectomy [1], with prior surgical experience suggesting a threshold of around 30 mm [11]. It remains unclear whether endocardial septal ablation—a procedure that addresses only the septal component of the obstruction—encounters a similar ‘anatomical ceiling’ in patients with excessively long leaflets.

Currently, patient selection criteria for PESA are not fully established, particularly concerning anatomical predictors of success. Furthermore, ablation strategies regarding the extent of the target zone (pure SAM zone vs. expanded zones) vary across centers [12]. This study aimed to analyze the correlation between preoperative echocardiographic characteristics and post-ablation outcomes, specifically investigating the predictive value of AMLL, and to summarize our center’s experience with a “Pan-SAM zone” ablation strategy.

## 2. Materials and Methods

### 2.1. Study Population

This single-center, retrospective study analyzed clinical and echocardiographic data from 30 consecutive patients with symptomatic HOCM who underwent ICE-guided PESA at the Department of Cardiology, Zhongshan Hospital, Fudan University, between February 2021 and August 2022. Baseline and procedural data were collected from medical records, and follow-up echocardiography was performed 1 year after the procedure.

Inclusion criteria were as follows: patients with a confirmed diagnosis of hypertrophic obstructive cardiomyopathy (HOCM); persistent symptoms related to left ventricular outflow tract obstruction despite at least 3 months of optimal medical therapy, including exertional dyspnea, chest pain, palpitations, dizziness, or syncope; a resting left ventricular outflow tract gradient (LVOTG) ≥ 50 mmHg on transthoracic echocardiography (TTE) accompanied by systolic anterior motion (SAM) of the mitral valve; and age ≥ 18 years.

Exclusion criteria were: pure apical hypertrophic cardiomyopathy; non-obstructive hypertrophic cardiomyopathy; a resting LVOTG < 50 mmHg on TTE; a history of prior surgical septal myectomy or transcatheter alcohol septal ablation; concomitant cardiac conditions requiring surgical intervention (such as severe mitral valve disease or multivessel coronary artery disease); or a left ventricular ejection fraction (LVEF) < 40% or New York Heart Association (NYHA) functional class IV, indicating an inability to tolerate the procedure.

### 2.2. Echocardiographic Assessment

Transthoracic echocardiography (TTE) was performed at baseline and the 1-year follow-up. Key measurements included: (1) Anterior mitral leaflet length (AMLL): AMLL was measured in the parasternal long-axis view during mid-diastole, from the hinge point of the mitral annulus to the leaflet tip, following the curvature of the leaflet. Measurements from three consecutive cardiac cycles were averaged for each patient. If image quality was suboptimal, the measurement was repeated in the apical long-axis view. (2) Interventricular septal thickness (IVST): Maximum thickness of the basal septum at end-diastole. (3) Hypertrophy distribution: Classified as basal only, basal + middle, or basal + middle + apical. (4) LVOTG: Peak gradient measured at rest. Resting LVOTG was measured using continuous-wave Doppler in the apical five-chamber view. For inclusion in the study, patients were required to have a baseline resting LVOTG ≥ 50 mmHg. To maximize comparability, resting LVOTG at baseline and follow-up was assessed under similar resting clinical conditions (i.e., without provocation and on stable medical therapy whenever possible). Follow-up TTE was performed at 12 months (± 30 days) after the procedure. All pre- and post-procedural transthoracic echocardiographic images were acquired by a single experienced echocardiologist (>10 years of experience) to minimize measurement variability; formal inter- and intra-observer reproducibility was not separately quantified.

### 2.3. Ablation Procedure

At our center, ICE-guided PESA was performed using a modified ablation strategy termed the “Pan-SAM zone” approach. This strategy integrates systolic contact mapping with diastolic anatomical extension using the CARTO 3 system and SoundStar ICE catheter:

(1) Systolic mapping (the core zone): Systolic contact mapping was standardized under ICE guidance. The interventricular septum and anterior mitral leaflet were visualized in the long-axis view during mid-systole, timed by surface ECG. The precise point of maximal septal–leaflet contact—defined as the intersection of leaflet echogenicity with the septal endocardium and confirmed by color Doppler aliasing—was tagged on the CARTO 3 electroanatomical map as the ‘core obstruction zone’. To ensure consistency, mapping was performed during stable hemodynamic conditions, with at least three cardiac cycles evaluated before final tagging. When contact points shifted dynamically during systole, the most central location was designated as the primary landmark and proximal locations as secondary tags.

(2) Diastolic extension (the safety margin): The map was then reviewed in diastole. The ablation target area was extended by 5–10 mm basally and apically from the core zone tags, determined by anatomical boundaries identified on ICE long- and short-axis views and confined to the hypertrophied septum. This ‘diastolic extension’ accounts for the hyperdynamic longitudinal excursion of the septum during systole, ensuring that the entire myocardial segment responsible for obstruction is adequately covered even during vigorous contraction. This approach differs from strategies targeting only the static anatomical bulge or the systolic contact point alone.

Ablation was performed in power-controlled mode (35–40 W) with an irrigation flow rate of 17–30 mL/min. Adequate lesion formation at each ablation site was assessed using a combination of electrophysiological and imaging criteria, including: (1) a local impedance drop of >10 Ω; (2) a >50% reduction in bipolar electrogram amplitude; and (3) visualization on intracardiac echocardiography (ICE) of a distinct tissue edema zone characterized by increased echogenicity and focal swelling, with an adequate edema zone depth indicating sufficient lesion penetration.

The procedural endpoint was defined by a combination of anatomical and hemodynamic criteria: (1) completion of ablation within the predefined obstructive target region, including the systolic anterior motion (SAM) contact zone and its adjacent extended areas (Pan-SAM zone); when direct ablation within the target region was limited by proximity to the conduction system, lesion delivery was cautiously expanded to surrounding septal areas deemed safe; and/or (2) an immediate invasive reduction in left ventricular outflow tract gradient (LVOTG) of ≥50% compared with baseline.

**Figure 1:**
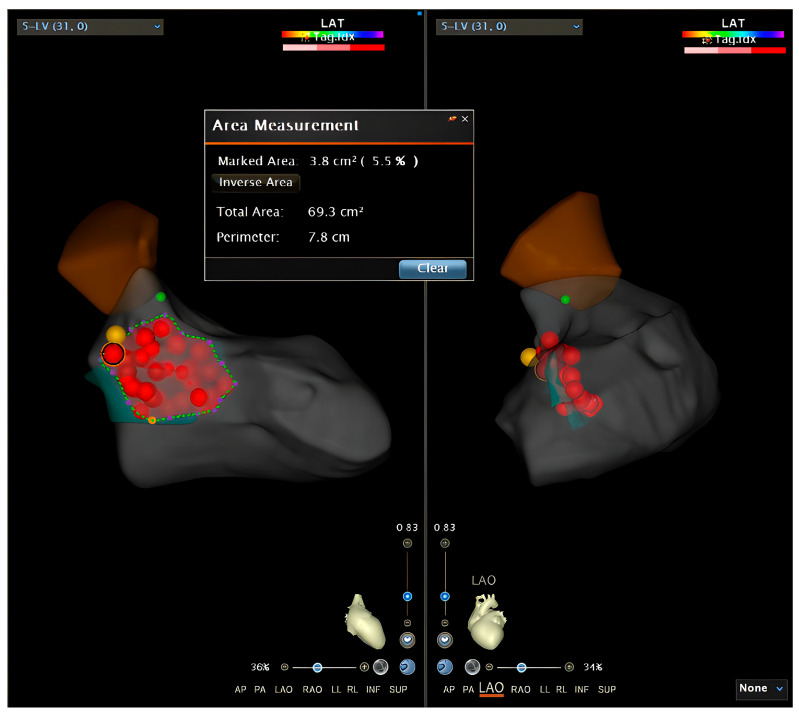
CARTO 3-based three-dimensional electroanatomical reconstruction of the left ventricle illustrating the Pan-SAM zone ablation strategy. The core zone was identified during systole based on septal–mitral leaflet contact and subsequently expanded basally and apically in diastole to form the Pan-SAM zone (outlined). Red tags indicate delivered radiofrequency ablation lesions. Yellow markers denote conduction system landmarks. In this representative case, the cumulative ablation area measured 3.8 cm^2^.

Radiofrequency ablation lesions were then delivered within the dynamically defined Pan-SAM zone (Figure 1), with a safety distance from the His bundle maintained by continuous intraprocedural electrogram monitoring.

All 30 procedures were performed by experienced electrophysiologists from our center’s cardiac electrophysiology team (>10 years of ablation experience), following a standardized study protocol to minimize inter-operator variability.

### 2.4. Statistical Analysis

Statistical analyses were performed using R (version 4.5.2). Continuous variables are summarized as mean ± SD or median (IQR), with normality assessed by the Shapiro–Wilk test. Between-group comparisons of continuous variables used Student’s *t*-test (normal distributions with equal variances by F-test), Welch’s *t*-test (normal distributions with unequal variances), or the Wilcoxon rank-sum test (non-normal distributions). Within-patient changes from baseline to 1 year were compared using the paired *t*-test or the Wilcoxon signed-rank test for continuous variables, and McNemar’s test for paired binary outcomes. Categorical variables between independent groups were compared using Fisher’s exact test. The association between AMLL and absolute LVOTG reduction was assessed by linear regression, with MM-estimator robust regression as a sensitivity analysis. Predictors of the binary secondary endpoint (≥50% LVOTG reduction) were evaluated by logistic regression; given the limited sample size (*n* = 30, with 10 patients in the smaller outcome category), the multivariable model was pre-specified to include only AMLL and basal-only hypertrophy. To address the limited sample size and the small number of patients in the non-significant response group, Firth penalized logistic regression was performed as a bias-reduction sensitivity analysis for the prespecified multivariable logistic model. Ridge logistic regression was additionally explored as a shrinkage-based sensitivity analysis to assess the directional stability of the association between AMLL and significant LVOTG reduction. Multicollinearity among candidate predictors was assessed using variance inflation factors (VIFs) and Spearman correlation analysis. Model calibration was summarized using the Brier score; given the limited sample size, formal calibration testing (e.g., calibration slope/intercept) was regarded as unreliable, and the model is presented as exploratory. Discriminative performance of AMLL was assessed by ROC analysis (DeLong 95% CI for AUC), and the stability of the Youden-index optimal cutoff was assessed by 1000 bootstrap replicates. A two-sided *p* < 0.05 was considered significant.

## 3. Results

### 3.1. Baseline Characteristics and Overall Efficacy

The baseline clinical and echocardiographic characteristics of the 30 patients are summarized in Table 1. The cohort included 30 patients (mean age 62.10 ± 9.81 years; 46.7% male). Baseline TTE showed a median IVST of 16.00 mm (IQR 15.00–19.75) and a mean AMLL of 27.53 ± 3.22 mm.

At the 1-year follow-up, the mean resting LVOTG decreased significantly from 86.03 ± 24.30 mmHg to 41.43 ± 18.49 mmHg, corresponding to a mean reduction of 51.88 ± 16.82% (*p* < 0.001). The proportion of patients with exertional dyspnea or chest pain decreased from 29/30 (96.7%) at baseline to 7/30 (23.3%) at 1 year (*p* < 0.001). NYHA functional class improved from 2.83 ± 0.46 at baseline to 1.70 ± 0.53 at 1 year (*p* < 0.001).

### 3.2. Predictors of Significant LVOTG Reduction (Secondary Endpoint)

For the key secondary endpoint, patients were classified as having significant efficacy (≥50% reduction in resting LVOTG, *n* = 20) or non-significant efficacy (<50%, *n* = 10). Baseline characteristics of the two groups are compared in Table 2.

The univariable and multivariable logistic regression results are shown in Table 3. In the prespecified multivariable logistic model adjusted for basal-only septal hypertrophy, AMLL was inversely associated with significant LVOTG reduction (OR per 1 mm increase: 0.429; 95% CI: 0.199–0.704; *p* = 0.006). Although basal-only septal hypertrophy was associated with the outcome in the univariable analysis (OR: 5.444; 95% CI: 1.115–32.874; *p* = 0.045), it was not independently associated with the endpoint after adjustment (OR: 1.306; 95% CI: 0.109–14.118; *p* = 0.822). Baseline IVST showed a non-significant trend (*p* = 0.052) in the unadjusted between-group comparison (Table 2), suggesting that massive septal thickness may pose a challenge to standard ablation energy penetration.

Given the limited sample size, sensitivity analyses were performed to evaluate the robustness of the logistic regression findings. In the Firth penalized logistic regression, AMLL remained inversely associated with significant LVOTG reduction in the multivariable model (OR per 1 mm increase: 0.510; 95% CI: 0.272–0.772; *p* < 0.001), whereas basal-only septal hypertrophy did not remain significant after adjustment (OR: 1.278; 95% CI: 0.142–10.202; *p* = 0.815). Ridge logistic regression yielded a consistent negative coefficient for AMLL under both lambda.min and lambda.1se conditions, supporting the directional stability of this association under coefficient shrinkage. No substantial multicollinearity was detected in the prespecified multivariable model, with VIFs of 1.024 for both AMLL and basal-only hypertrophy. In the broader candidate predictor set, including AMLL, IVST, baseline LVOTG, and basal-only hypertrophy, all VIFs were below 2, and Spearman correlations among these predictors were weak to moderate (all |ρ| ≤ 0.52). Detailed results of the penalized regression sensitivity analyses, multicollinearity diagnostics, and model calibration are summarized in Supplementary Table S1.

**Figure 2:**
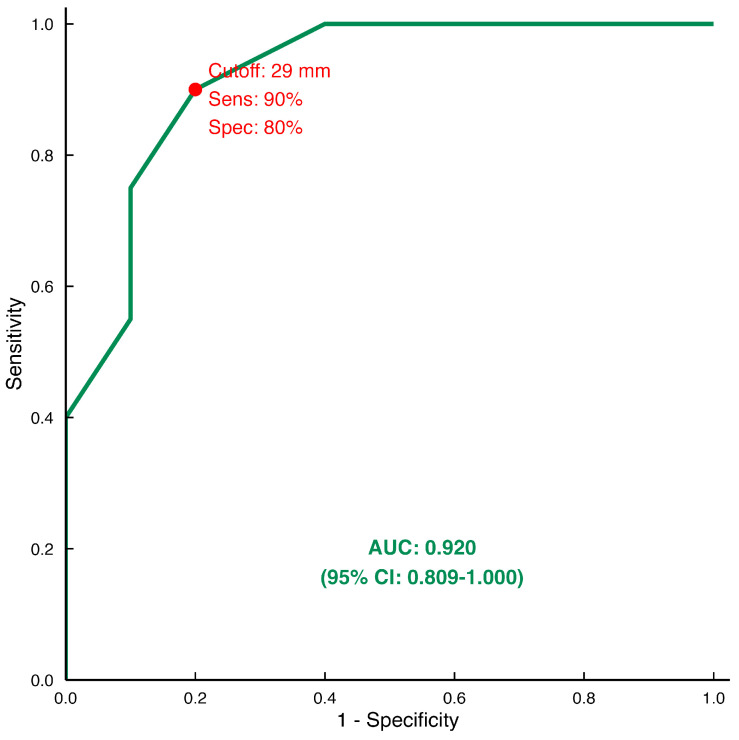
Receiver operating characteristic (ROC) curve of anterior mitral leaflet length (AMLL) for predicting significant LVOTG reduction.

As shown in Figure 2, the ROC curve evaluates the performance of AMLL in predicting significant procedural efficacy (≥50% LVOTG reduction). The AUC was 0.920 (95% CI: 0.809–1.000; *p* < 0.001). The Youden index identified an exploratory cutoff value of approximately 29 mm, yielding a sensitivity of 90% and a specificity of 80%. Bootstrap resampling (1000 replicates; Figure S1) supported the stability of this 29 mm cutoff, with this value being the most frequently identified threshold across resamples. This Youden-derived threshold should be interpreted as exploratory and requires validation in larger independent cohorts before clinical application.

**Figure 3:**
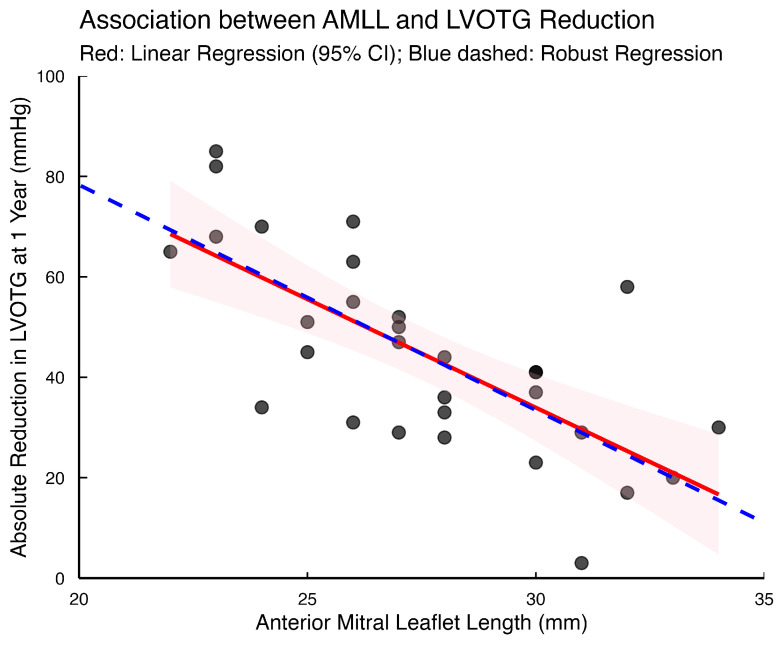
Association between anterior mitral leaflet length (AMLL) and absolute reduction in resting LVOT gradient (LVOTG) at the 1-year follow-up. Each dot represents one patient. The solid red line shows the fitted linear regression with a 95% confidence interval (shaded). The blue dashed line indicates robust regression (MM-estimator) as a sensitivity analysis to reduce the influence of potential outliers. LVOTG reduction was calculated as baseline resting LVOTG minus the 1-year resting LVOTG. Linear regression: β = −4.32 mmHg per mm (95% CI: −5.99 to −2.65; *p* < 0.001; *R*^2^ = 0.50).

As a continuous-outcome analysis, AMLL was also inversely associated with the absolute reduction in resting LVOTG at 1 year (Figure 3). Each 1 mm increase in AMLL corresponded to approximately 4.3 mmHg less LVOTG reduction (β = −4.32, 95% CI: −5.99 to −2.65; *p* < 0.001), and AMLL alone explained approximately half of the variance in gradient reduction (*R*^2^ = 0.50). The association remained robust in the MM-estimator sensitivity analysis (β = −4.49, 95% CI: −6.37 to −2.60; *p* < 0.001).

### 3.3. Procedural Parameters and Safety

Key intraprocedural ablation metrics are summarized in Table 4. The mean cumulative ablation time was 18.16 ± 4.97 min, with a mean power of 36.80 ± 4.64 W and a mean impedance drop of 12.88 ± 2.48 Ω. The Pan-SAM zone strategy resulted in a mean actual ablation area of 3.76 ± 0.83 cm^2^, substantially exceeding the mapped SAM-contact area (2.34 ± 0.50 cm^2^), reflecting the deliberate diastolic extension of the target zone. The mean ablation depth was 4.77 ± 1.07 mm.

**Figure 4:**
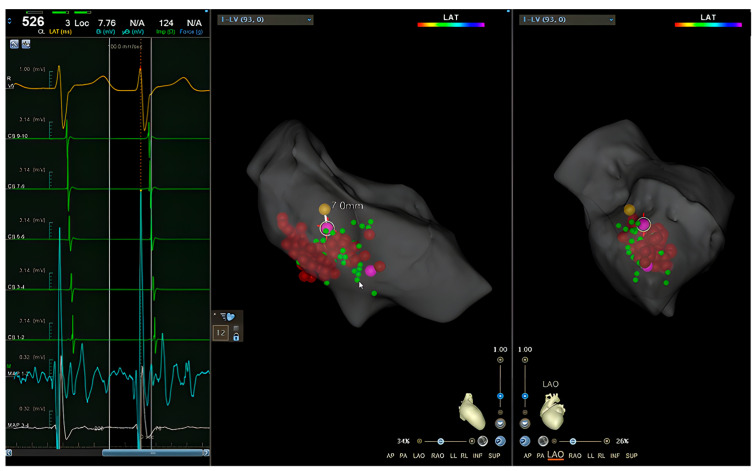
The His bundle–ablation site spatial relationship and safety considerations. (**Left**) Intracardiac electrograms showing the His bundle potential (yellow tag) and the ablation site (pink tag). (**Middle/Right**) 3D mapping views showing the 7.0 mm distance. Energy delivery at this site induced a transient accelerated junctional rhythm, highlighting the need for continuous electrogram monitoring regardless of anatomical distance.

The anatomical distance from the nearest effective ablation lesion to the tagged His bundle–branch potential was measured in all 30 patients. The shortest distance was 3.7 mm, the longest was 7.8 mm, and the mean was 6.56 ± 1.04 mm. In one patient, energy delivery 7.0 mm from the recorded His potential induced a transient accelerated junctional rhythm that resolved immediately upon cessation of energy (Figure 4). This observation indicates that anatomical distance alone is insufficient to define a strict safety threshold and should be interpreted together with real-time electrogram monitoring—specifically, the absence of any His/branch signal recorded by the ablation catheter at the intended target site. Post-ablation ICE imaging confirmed effective lesion formation in all patients, and no patient developed permanent atrioventricular block, pericardial complications, or procedure-related death during the 1-year follow-up.

## 4. Discussion

This study demonstrates that ICE-guided PESA is an effective strategy for relieving left ventricular outflow tract obstruction (LVOTO) in patients with hypertrophic obstructive cardiomyopathy. Notably, our findings also highlight that the underlying anatomical substrate strongly influences the efficacy of this approach. Among the evaluated echocardiographic parameters, anterior mitral leaflet length (AMLL) emerged as an independent echocardiographic correlate of procedural response in the prespecified multivariable model, underscoring the central role of mitral valve morphology in modulating the response to septal ablation.

### 4.1. Anatomical Constraint Imposed by AMLL

Our finding that AMLL was independently associated with procedural outcome in the prespecified model underscores its role in LVOTO pathophysiology. The pathophysiology of LVOTO in hypertrophic cardiomyopathy is fundamentally governed by hydrodynamic drag forces acting on the anterior mitral leaflet, which draw the leaflet toward the interventricular septum and sustain systolic anterior motion [13]. Elongation of the anterior mitral leaflet, a common phenotypic feature in this disease, allows the leaflet to protrude further into the outflow tract, thereby increasing its exposed surface area and amplifying the drag force generated by systolic ejection flow [14].

Surgical experience has long recognized mitral valve morphology as a key determinant of LVOTO, with concomitant mitral valve intervention recommended when AMLL exceeds approximately 30 mm [11]. The 29 mm cutoff in our cohort closely approaches this surgical threshold, suggesting a shared “anatomical ceiling” for septal reduction therapies, where the valvular contribution becomes increasingly dominant.

Among alternatives to septal myectomy, alcohol septal ablation (ASA) is the most widely adopted percutaneous approach, yet its efficacy is constrained by coronary anatomy and septal perforator distribution. Emerging evidence has further shown that redundant AMLL is associated with suboptimal ASA outcomes [15], highlighting the importance of mitral valve phenotyping in patient selection. Our finding that AMLL constrains PESA efficacy extends this concept to the endocardial ablation domain. Collectively, these data support the integration of pre-procedural anatomical phenotyping into therapeutic strategy selection across all septal reduction modalities. In such settings, even extensive septal functional modification may fail to fully counterbalance the persistent drag imposed by a redundant anterior mitral leaflet, resulting in ongoing systolic anterior motion–septal interaction and attenuated LVOT gradient reduction. Rather than representing a discrete threshold phenomenon, the influence of AMLL on procedural outcome appears to follow a continuum. When leaflet length is relatively modest, LVOTO seems to be driven predominantly by septal hypertrophy, and functional modification of the septal contribution alone is often sufficient to achieve meaningful relief of LVOT obstruction. As leaflet elongation becomes more marked, however, the mitral valve apparatus contributes progressively to the obstructive physiology, and septal ablation alone may no longer provide complete or durable relief. In this context, AMLL functions not merely as a descriptive anatomical parameter but as an integrative marker of the relative contributions of septal and valvular mechanisms to LVOTO.

We also observed a non-significant trend (*p* = 0.052) suggesting that greater interventricular septal thickness may be associated with less optimal gradient reduction. Given a mean ablation lesion depth of approximately 4.8 mm, it is plausible that, in patients with severe hypertrophy, the induced lesion volume represents a relatively smaller proportion of the total septal mass. In such cases, standard ablation energy may be insufficient to induce the necessary morphological remodeling to fully relieve the obstruction, highlighting a potential ‘thickness-dependent’ efficacy limit similar to the ‘length-dependent’ limit imposed by the AMLL.

It is important to emphasize that the 29 mm threshold identified in this single-center cohort is exploratory. While it closely approximates the surgical threshold of ~30 mm, external validation in multicenter studies is essential before this parameter can be incorporated into clinical decision-making algorithms.

### 4.2. Ablation Strategy: Expanding the Target While Respecting Safety

Incomplete or insufficient lesion formation has been recognized as an important cause of residual obstruction or recurrence after septal ablation. A review of prior single-center studies reveals substantial heterogeneity in ablation endpoints and lesion extent, with reported target areas ranging from focal SAM-contact ablation to extensive basal septal modification [4,16,17,18]. While several studies suggest that broader lesion coverage may be associated with greater LVOT gradient reduction, excessive ablation—particularly toward the basal septum—inevitably raises concerns regarding procedural complexity and conduction system injury [2,3].

To address the dynamic and spatially complex nature of SAM-related obstruction, we adopted a “Pan-SAM zone” strategy that integrates systolic functional mapping with diastolic anatomical extension. Rather than targeting the static anatomical bulge or the systolic contact point alone, this approach aims to modify the entire myocardial substrate that contributes to obstruction throughout the cardiac cycle. Unlike alcohol septal ablation, which is anatomically constrained by septal perforator distribution [19], ICE-guided PESA allows for precise, contiguous lesion creation independent of vascular anatomy, thereby facilitating this comprehensive ‘Pan-SAM’ coverage.

In limited single-center ICE-guided PESA series [2,4,5,16,17,20], ablation endpoints vary considerably regarding the extent of SAM-zone coverage. While broader lesion coverage appears to correlate with greater LVOTG reduction, excessive extension inevitably increases procedural risk. A quantitative comparison of our cohort with previously published single-center ICE-guided PESA series is provided in Supplementary Table S2. To balance efficacy and safety, we adopted a “Pan-SAM zone” strategy that integrates systolic contact mapping with diastolic anatomical extension. Using this strategy, we achieved a mean ablation area of 3.76 cm^2^, exceeding the mapped systolic SAM-contact area (2.34 cm^2^), with a corresponding mean LVOT gradient reduction of approximately 52% at the 1-year follow-up. Importantly, this expansion of lesion extent did not compromise procedural safety: no permanent atrioventricular block, pericardial complication, or procedure-related death occurred during the 1-year follow-up. A single episode of transient junctional rhythm occurred 7.0 mm from the tagged His potential, underscoring the need for continuous electrogram surveillance regardless of anatomical distance. However, given the limited sample size (*n* = 30) and follow-up duration, these safety data should be regarded as preliminary. The true incidence of rare complications such as permanent conduction block may be underestimated, and longer follow-up in larger cohorts is needed to establish the definitive safety profile of this technique.

Collectively, these findings suggest that the Pan-SAM zone strategy may represent a balanced and reproducible approach, maximizing functional efficacy while respecting anatomical safety margins. However, direct head-to-head comparative studies are needed to determine whether it yields greater gradient reduction than more limited ablation strategies.

### 4.3. Technical Considerations and Catheter Selection

Technical maneuverability remains an important determinant of procedural success in PESA, particularly when targeting the basal septum via a retrograde aortic approach. Although contact-force sensing catheters provide valuable real-time feedback, their stiffer, sensor-equipped tips may limit extreme deflection and stable contact in certain anatomical configurations. In one patient with severe basal septal hypertrophy, the contact-force catheter could not be positioned reliably because of the acute angle required. Transitioning to a standard irrigated catheter with a shorter and more flexible distal curve allowed stable contact and effective energy delivery.

This experience highlights the need for procedural flexibility and individualized tool selection. Adapting catheter choice to septal geometry and access angle may be essential for achieving adequate lesion formation, particularly in patients with marked basal hypertrophy.

### 4.4. Limitations

Several limitations of this study should be acknowledged. First, the retrospective, single-center design and relatively small sample size limit the generalizability of the findings and reduce the statistical power of multivariable analyses. With an events-per-variable ratio of 5 for the binary secondary endpoint, the corresponding odds ratio confidence interval is wide; the primary continuous-endpoint analysis was nonetheless well powered (post hoc power > 99%). Second, the follow-up duration was limited to one year, and the longer-term durability of gradient reduction remains to be established. Third, septal thickness (IVST) showed a borderline association (*p* = 0.052) with LVOT gradient reduction that did not reach statistical significance, likely due to the limited sample size; this finding should be regarded as hypothesis-generating. Fourth, although bootstrap resampling and penalized regression sensitivity analyses supported the internal stability of the findings, the identified AMLL cutoff of 29 mm was derived from a small single-center cohort. Prospective multicenter validation is therefore required before this threshold can be incorporated into clinical decision-making. Fifth, for patient safety, only resting LVOTG was assessed; exercise or Valsalva provocation was not performed given the risk of exacerbating outflow obstruction in patients with high baseline gradients. Consequently, we cannot determine whether PESA differentially affects resting versus provocable obstruction. Future studies will incorporate standardized exercise echocardiography to comprehensively evaluate the hemodynamic response to PESA.

## 5. Conclusions

ICE-guided endocardial septal ablation, using a Pan-SAM zone strategy, provides effective gradient reduction in HOCM with a favorable preliminary conduction-safety profile. Anterior mitral leaflet length emerged as the main independent anatomical correlate of hemodynamic response in this cohort: a Youden-derived exploratory cutoff of approximately 29 mm may help identify patients who are more likely to achieve substantial gradient reduction, whereas those with longer leaflets may warrant individualized consideration of more extensive ablation or adjunctive mitral valve strategies. External validation in larger, multicenter cohorts is needed before this threshold is applied in clinical decision-making.

## Figures and Tables

**Table 1:** Baseline clinical and transthoracic echocardiographic characteristics.

Variable	Value
Number of patients	30
Male sex, *n* (%)	14 (46.7)
Age, years	62.10 ± 9.81
Body mass index, kg/m^2^	24.95 ± 2.60
Heart rate, bpm	63.73 ± 5.25
Systolic blood pressure, mmHg	122.37 ± 10.84
Diastolic blood pressure, mmHg	72.43 ± 9.52
Hypertension, *n* (%)	14 (46.7)
Diabetes mellitus, *n* (%)	4 (13.3)
Paroxysmal atrial fibrillation, *n* (%)	4 (13.3)
NYHA functional class III, *n* (%)	24 (80.0)
Baseline medications, *n* (%)	
β-blockers	30 (100.0)
Non-dihydropyridine CCBs	14 (46.7)
Transthoracic echocardiography	
LAD, mm	45.23 ± 6.08
LVEDD, mm	44.77 ± 3.56
LVESD, mm	28.03 ± 2.70
LVEF, %	66.97 ± 4.73
AMLL, mm	27.53 ± 3.22
IVST, mm	16.00 (15.00, 19.75)
Septal hypertrophy distribution, *n* (%)	
Basal segment only	17 (56.7)
Basal + middle segments	7 (23.3)
Basal + middle + apical segments	6 (20.0)
Preoperative LVOTG, mmHg	86.03 ± 24.30
LVOTG at 1-year follow-up, mmHg	41.43 ± 18.49
Percentage reduction in LVOTG, %	51.88 ± 16.82

Data are presented as mean ± SD, median (interquartile range), or n (%). AMLL = anterior mitral leaflet length; CCBs = calcium channel blockers; IVST = interventricular septal thickness at end-diastole; LAD = left atrial diameter; LVEDD = left ventricular end-diastolic diameter; LVEF = left ventricular ejection fraction; LVESD = left ventricular end-systolic diameter; LVOTG = left ventricular outflow tract gradient; NYHA = New York Heart Association.

**Table 2:** Comparison between patients with significant and non-significant LVOTG reduction.

Variable	Significant Reduction (*n* = 20)	Non-Significant Reduction (*n* = 10)	*p* Value
Male sex, n (%)	10 (50.0)	4 (40.0)	0.709
Age, years	62.25 ± 10.67	61.80 ± 8.35	0.908
Hypertension, n (%)	7 (35.0)	7 (70.0)	0.122
LAD, mm	45.30 ± 6.51	45.10 ± 5.45	0.934
LVEDD, mm	44.60 ± 3.47	45.10 ± 3.90	0.724
IVST, mm	15.50 (15.00, 16.75)	16.50 (16.00, 21.00)	0.052
LVEF, %	66.85 ± 5.45	67.20 ± 3.05	0.852
Baseline LVOTG, mmHg	83.65 ± 25.93	90.80 ± 21.18	0.457
AMLL, mm	25.95 ± 2.31	30.70 ± 2.36	<0.001
Basal septal hypertrophy only, n (%)	14 (70.0)	3 (30.0)	0.056

LAD, left atrial diameter; LVEDD, left ventricular end-diastolic diameter; IVST, interventricular septal thickness; LVEF, left ventricular ejection fraction; LVOTG, left ventricular outflow tract gradient; AMLL, anterior mitral leaflet length.

**Table 3:** Logistic regression analysis of factors associated with significant LVOTG reduction.

Variable	Category	Univariable OR (95% CI)	*p* Value	Multivariable OR (95% CI)	*p* Value
AMLL (per mm)	—	0.423 (0.198–0.680)	0.005	0.429 (0.199–0.704)	0.006
Basal septal hypertrophy only	Yes	5.444 (1.115–32.874)	0.045	1.306 (0.109–14.118)	0.822
	No	Reference	—	Reference	—

**Table 4:** Procedural and ablation parameters.

Parameter	Value (*n* = 30)
Mean cumulative ablation time (min)	18.16 ± 4.97
Mean power (W)	36.80 ± 4.64
Impedance drop (Ω)	12.88 ± 2.48
Mapped SAM area (cm^2^)	2.34 ± 0.50
Total ablation area (cm^2^)	3.76 ± 0.83
Edema zone depth (mm)	4.77 ± 1.07

SAM = systolic anterior motion.

## Data Availability

The raw patient-level data are available from the corresponding author upon reasonable request, subject to institutional ethics approval.

## Supplementary Materials

The following supporting information can be downloaded at: https://www.mdpi.com/article/10.3390/jcdd13060274/s1. Figure S1: Distribution of the Youden-derived AMLL cutoff from 1000 bootstrap replicates; Table S1: Penalized logistic regression sensitivity analyses and model diagnostics; Table S2: Comparison of ICE-guided PESA outcomes in published single-center series (2016–2026).

## Abbreviations

The following abbreviations are used in this manuscript:

3D	three-dimensional
AMLL	anterior mitral leaflet length
ASA	alcohol septal ablation
AUC	area under the curve
CI	confidence interval
HCM	hypertrophic cardiomyopathy
HOCM	hypertrophic obstructive cardiomyopathy
ICE	intracardiac echocardiography
IVST	interventricular septal thickness
LAD	left atrial diameter
LVEDD	left ventricular end-diastolic diameter
LVEF	left ventricular ejection fraction
LVOTO	left ventricular outflow tract obstruction
LVOTG	left ventricular outflow tract gradient
NYHA	New York Heart Association
OR	odds ratio
PESA	percutaneous endocardial septal ablation
ROC	receiver operating characteristic
SAM	systolic anterior motion
SD	standard deviation
TTE	transthoracic echocardiography
VIF	variance inflation factor
