# Supplementary material for: Impact of Anterior Mitral Leaflet Length on the Efficacy of Intracardiac Echocardiography-Guided Endocardial Septal Ablation for HOCM
Source: J Cardiovasc Dev Dis. 2026 Jun 16;13(6):274. doi: 10.3390/jcdd13060274 (PMC13301077; doi:10.3390/jcdd13060274)
Supplement: Supplementary file 1 [file jcdd-13-00274-s001.zip › jcdd-4311809-supplementary.pdf]

Supplementary Table S1. Penalized logistic regression sensitivity analyses and model diagnostics

Model / Analysis	Variable	Estimate	95% CI	*P* value
**A. Firth penalized logistic regression**				
Univariable	AMLL	OR 0.489	0.254–0.735	<0.001
Univariable	Basal-only hypertrophy	OR 4.780	1.053–25.748	0.043
Multivariable	AMLL	OR 0.510	0.272–0.772	<0.001
Multivariable	Basal-only hypertrophy	OR 1.278	0.142–10.202	0.815
**B. Ridge logistic regression (shrinkage estimates)**				
λ.min	AMLL	OR 0.642	—	—
λ.min	Basal-only hypertrophy	OR 1.622	—	—
λ.1se	AMLL	OR 0.854	—	—
λ.1se	Basal-only hypertrophy	OR 1.486	—	—
**C. Multicollinearity (variance inflation factor)**				
Main model	AMLL	1.024	—	—
Main model	Basal-only hypertrophy	1.024	—	—
Candidate set	AMLL	1.461	—	—
Candidate set	IVST	1.333	—	—
Candidate set	Baseline LVOTG	1.090	—	—
Candidate set	Basal-only hypertrophy	1.631	—	—
**D. Calibration (Brier score)**				
Standard logistic model	Brier score	0.098	—	—
Firth logistic model	Brier score	0.099	—	—
**E. Spearman correlation (candidate predictors)**				
Pairwise ϱ	AMLL – IVST	0.50	—	—
Pairwise ϱ	AMLL – Baseline LVOTG	−0.25	—	—
Pairwise ϱ	AMLL – Basal-only hypertrophy	−0.52	—	—
Pairwise ϱ	IVST – Baseline LVOTG	−0.13	—	—
Pairwise ϱ	IVST – Basal-only hypertrophy	−0.52	—	—
Pairwise ϱ	Baseline LVOTG – Basal-only hypertrophy	0.05	—	—

OR = odds ratio; CI = confidence interval; AMLL = anterior mitral leaflet length; IVST = interventricular septal thickness; LVOTG = left ventricular outflow tract gradient; VIF = variance inflation factor; ϱ = Spearman correlation coefficient; λ = ridge penalization parameter (lambda); — denotes not applicable. Firth estimates are penalized maximum-likelihood odds ratios obtained to reduce small-sample bias. Ridge logistic regression provides shrinkage estimates only and does not yield conventional confidence intervals or P values; the consistently negative direction of the AMLL coefficient under both λ.min and λ.1se supports the stability of the association. VIF values below 2 and all pairwise |ϱ| ≤ 0.52 indicate no meaningful multicollinearity. The Brier score summarizes overall model performance (range 0–1, lower indicating better performance); given the limited sample size, formal calibration testing was considered unreliable and the model is presented as exploratory.

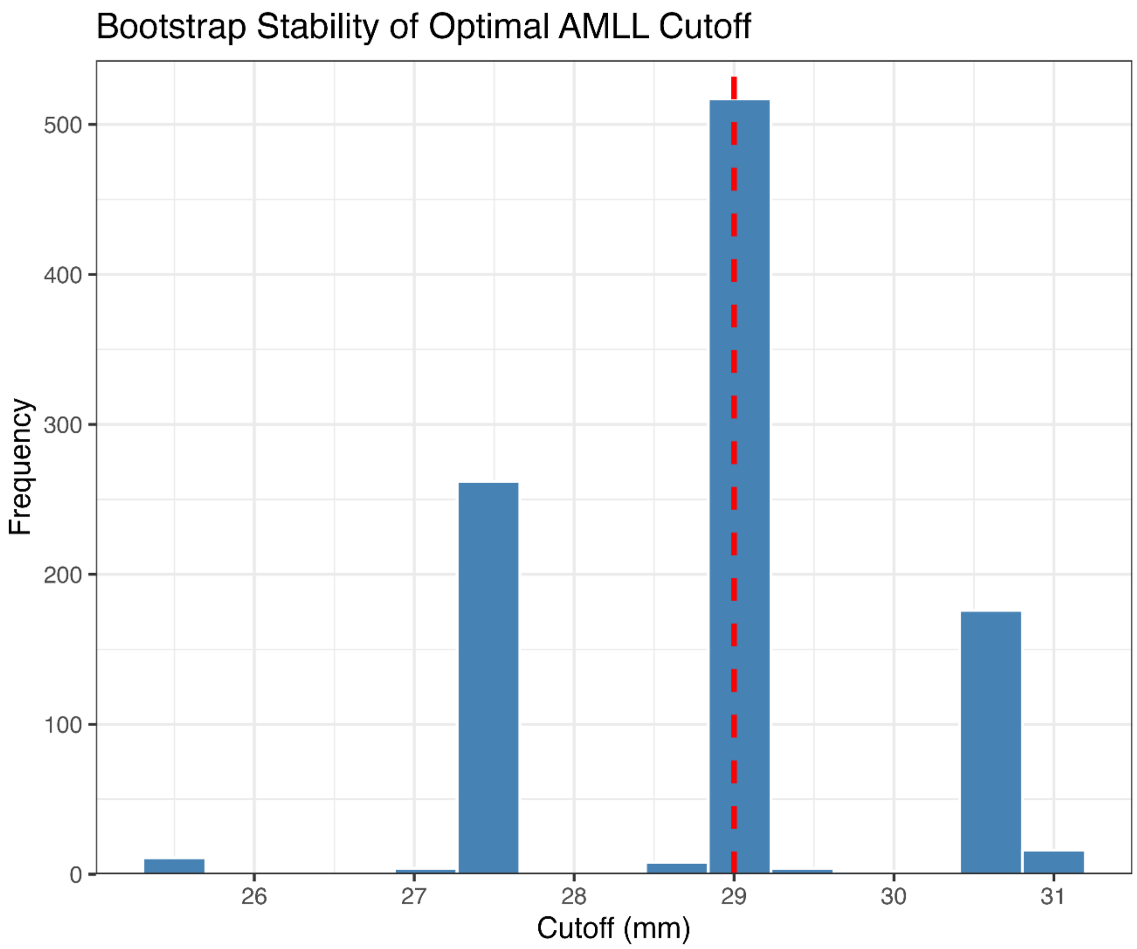

Figure S1. Distribution of the Youden-derived AMLL cutoff from 1000 bootstrap replicates. The red dashed line indicates the cutoff of 29 mm identified in the original cohort. The histogram illustrates the internal bootstrap distribution of this threshold across resampled datasets.

Supplementary Table S2. Comparison of ICE-guided PESA outcomes in published single-center series (2016–2026)

Study	N	Design	Mean age, years	Baseline resting LVOTG, mmHg	Post-procedure resting LVOTG, mmHg	LVOTG reduction, %	Ablation area, cm^2	Major complications	Follow-up
Present study	30	Retrospective	62.10 ± 9.81	86.03 ± 24.30	41.43 ± 18.49	51.88	3.76 ± 0.83	None severe	12 months
Zheng et al., 2025 [5]	20	Retrospective	56.29 ± 8.13	73.00 ± 25.47	33.25 ± 16.28	54	NR	None severe	2 years
Tian et al. (BMC), 2025 [17]	25	Retrospective	55.3 ± 13.5	79.0 ± 37.6	55.6 ± 34.8	29.6	NR (conservative, ~50% of obstructive area)	None severe	37 (25–47.5) months
Tian et al. (Ann Med Surg), 2024 [16]	19	Retrospective	54.8 ± 13.7	67.6 ± 34.2	52.3 ± 45	22.6	NR	None severe	3 (3–5.5) months
Guo et al., 2022 [20]	9	Retrospective	51.7 ± 12.2	85.9 ± 26.7	44.0 ± 15.0	48.7	NR	None severe	4 (3–6) months
Liu et al., 2021 [4]	20	Consecutive enrollment	57.7 ± 14.4	86.5 ± 29.2	48.1 ± 29.7	47	2.9 ± 1.4	None severe	6 months
Cooper et al., 2016 [2]	5	Prospective case series	59.2 (44–79)	64.2 ± 50.6	12.3 ± 2.5	81	14.6 (7.5–23.1)	1 death (retroperitoneal hemorrhage); 1 late complete AVB (at 6 month)	6 months

AVB = atrioventricular block. ICE = intracardiac echocardiography; NR = not reported; PESA = percutaneous endocardial septal radiofrequency ablation.
